# The associations between dynapenic obesity and cognitive disorder in older hypertension patients: a multicenter cross-sectional study

**DOI:** 10.3389/fpubh.2026.1893791

**Published:** 2026-09-29

**Authors:** Pingping An, Juxiao Wu, Yijing Zhang, Zhanlei Shen, Yudong Miao, Jian Wu, Quanman Li

**Affiliations:** 1 Department of Health Policy and Management, College of Public Health, Zhengzhou University, Henan, China; 2 Krieger School of Arts and Sciences, Johns Hopkins University, Baltimore, MD, United States

**Keywords:** cognitive disorder, depressive disorders, dynapenic obesity, hypertension, older adults, path analysis, physical activity

## Abstract

**Background:**

Evidence regarding the associations between dynapenic obesity and cognitive disorder in older adults is limited. This study aimed to investigate the prevalence of cognitive disorder among the older patients with hypertension (HTN), explore the association between dynapenic obesity and cognitive disorder, and explore a hypothesized path structure.

**Methods:**

We conducted a multicenter cross-sectional study from July 2023 to March 2024 among the older HTN patients aged 65 years and older. Data were collected using a structured questionnaire and standardized measurement. Logistic regression models were used to test the association between dynapenic obesity and cognitive disorder. Multiple imputation by chained equations under a missing-at-random assumption was conducted. Structural equation modeling was employed to explore the hypothesized path analysis. Subgroup and sensitivity analyses were performed to validate the robustness of the findings.

**Results:**

The study included 9,568 older HTN patients. The prevalence of cognitive disorder in this population was 15.67% (95% confidence interval [CI]: 14.94–16.40%). After adjusting for potential confounders, a significant positive association was observed between dynapenic obesity and cognitive disorder (adjusted odds ratio [aOR] = 1.418, 95% CI: 1.263–1.591). In the path model, dynapenic obesity was associated with lower odds of adequate physical activity (PA) (aOR = 0.708, 95% CI: 0.640–0.783). Adequate physical activity was associated with lower odds of depressive disorders (aOR = 0.441, 95% CI: 0.367–0.529) and cognitive disorder (aOR = 0.521, 95% CI: 0.452–0.601). Participants with depressive disorders had higher odds of cognitive disorder than those without depressive disorders (aOR = 1.312, 95% CI: 1.031–1.669). This association remained consistent across different age groups (all *p* < 0.05). Almost all results of sensitivity analysis were consistent with the main findings.

**Conclusion:**

Dynapenic obesity was positively associated with cognitive disorder. PA and depressive disorders were regarded as variables within a hypothesized cross-sectional path structure.

## Introduction

Recent decades have witnessed a significant increase in the global population of older adults. The global population of the older individuals is projected to double by 2050 (1). However, increased longevity does not necessarily correlate with improved health or quality of life. Despite extended lifespans, older adults remain vulnerable to chronic diseases and cognitive disorder (2, 3). Nevertheless, existing research on cognitive disorder has predominantly focused on their prevalence among healthy aging populations (4–6), often overlooking the compounding effects of chronic diseases. Few studies have systematically examined how comorbidities such as hypertension (HTN) may interact with neurodegenerative processes. This gap in the literature limits our understanding of the multifaceted risks faced by older adults with chronic diseases, who represent a growing yet understudied demographic.

Dynapenic obesity (also referred to as sarcopenic obesity), defined by the concurrent presence of sarcopenia and excessive adiposity, has emerged as an important area of research in recent years. Notably, obesity and muscle weakness have each been linked to cognitive functional impairment (7, 8), and recent studies have extended this literature to dynapenic or sarcopenic obesity. Contemporary studies have demonstrated that dynapenic obesity serves as a predictor of cognitive disorder (9, 10), suggesting a potential synergistic effect between adiposity and muscle dysfunction in neurological decline. A large community-based study found that the association varied by age group (11). Despite these important observations, the association between dynapenic obesity and functional impairments in patients with chronic diseases remains poorly understood, as current research has included patients with cardiometabolic disease (12), with limited studies addressing this critical research gap.

Substantial evidence has established the beneficial role of physical activity (PA) in promoting healthy aging. Notably, research demonstrates that regular PA may not only improve depressive disorders (13) but potentially prevent cognitive disorder (14, 15). Supporting evidence from the China Family Panel Studies, utilizing a cross-lagged design, indicates that depressive disorders often precede cognitive disorder in older adults (16), this association subsequently replicated in multiple independent studies (17–19). Current research has further elucidated the mediating pathways linking obesity and cognitive function. Studies have identified PA as a significant mediator in the obesity-cognitive disorder association (8), while other investigations have demonstrated depressive disorders as another critical mediating factor between obesity and cognitive disorder (20). However, despite these advances, the path of PA and depressive disorders in the association between dynapenic obesity (not general obesity) and cognitive disorder remain poorly understood, representing a critical gap in current knowledge.

In summary, we aim to examine the association between dynapenic obesity [operationally defined by handgrip strength and body mass index (BMI)] and cognitive disorder from a multicenter study. Moreover, we seek to elucidate the role of physical activity levels and depressive disorders in this prespecified cross-sectional path structure.

## Methods

### Study setting and participants

We conducted a baseline survey for the Northern China Lifestyle Medicine Cohort, a multicenter, observational study utilizing cluster random sampling, from July 1, 2023, to March 8, 2024 (registration number: ChiCTR2500090096200). The study was reported in accordance with the STROBE (Strengthening the Reporting of Observational Studies in Epidemiology) statement (Supplementary material). This survey targeted participants in Northern China, who were recruited through questionnaire interviews and physical measurements. Representative samples were obtained from the East, Central, West, and Northeast regions of Northern China, specifically from cities such as Ningjin (East), Jiaxian (Central), Delingha (West), and Linkou (Northeast), which were randomly selected from each respective region. Within each city, 2–4 towns were randomly chosen, and from these towns, villages/communities were further randomly selected. All individuals aged 65 years or older with HTN enrolled in the national basic public health service from the selected villages/communities were invited to participate. With the help of trained investigators, participants completed the survey questionnaire.

Initially, a total of 11,406 HTN patients from four geographic regions of Northern China were enrolled in the study. However, after excluding participants with missing data on depressive disorders (*n* = 86), sex (*n* = 6), body mass index (BMI) (*n* = 195), handgrip (*n* = 89), cognitive disorder (*n* = 1,401) and education level (*n* = 61), the final analysis included 9,568 older HTN patients. The minimum required sample size was estimated using the conventional formula for a cross-sectional study. Based on an expected prevalence of cognitive impairment of 15.3%, a 95% confidence level, a relative allowable error of 10% of the expected prevalence, a design effect of 2.0 for cluster sampling, and an anticipated nonresponse or invalid-data rate of 20%, the minimum required sample size was estimated to be 5,317 participants. The final analytical sample of 9,568 participants exceeded this requirement. A flowchart depicting the participant recruitment process is presented in Figure 1. The study was granted approval by the Ethics Committee of Zhengzhou University (ID: 2023–318), and all participants provided their written informed consent prior to enrollment.

**Figure 1:**
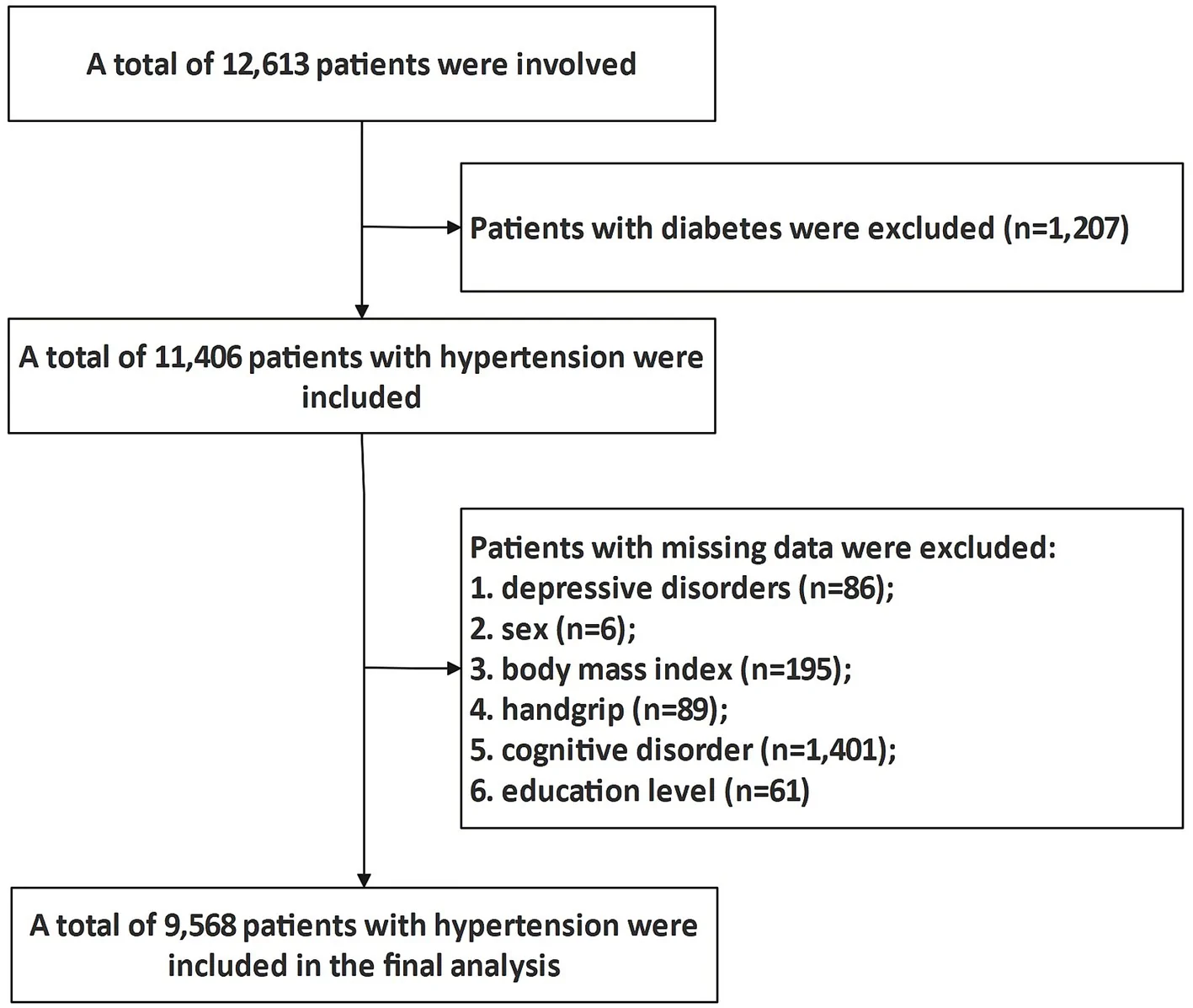
The flowchart of participants included in this study.

### Data collection and definitions

Data were gathered using a comprehensive questionnaire (21, 22) that covered various aspects, including socio-demographic characteristics, lifestyle factors, mental health conditions, and cognitive function. Socio-demographic variables included age, sex, marital status, education level, and so on. Additionally, lifestyle factors such as smoking and drinking habits, PA, and sleep duration were also assessed. PA was evaluated by considering the duration and frequency of light, moderate, and vigorous physical activities. Mental health conditions, particularly anxiety and depressive disorders, were assessed using validated tools. Cognitive function was evaluated using the Mini-Mental State Examination (MMSE), with cognitive disorder defined as an MMSE score below 17 for illiterate individuals, below 20 for those with primary education, and below 24 for those with secondary education or higher (23). A weighted weekly PA score was calculated as 2 * vigorous activity minutes plus moderate activity minutes, with adequate PA was defined as a score ≥150 min/week. Depressive disorders were assessed using the Patient Health Questionnaire-9 (PHQ-9), where a score of 7 or higher indicated clinically significant depressive disorders (24). Internal consistency was high for the anxiety items (Cronbach’s alpha = 0.911) and acceptable for the depression items (Cronbach’s alpha = 0.800).

We assessed the participants’ maximum handgrip strength by performing two measurements on each hand, and the highest value among the four measurements was used for analysis (25). Participants were classified as having sarcopenia if their handgrip strength was below 26 kg for the older men or 18 kg for the older women (26). Body height and weight were measured twice while participants wore light clothing and no shoes, and BMI was then calculated using the average of these measurements. Based on their BMI levels, participants were categorized as either normal weight (<24 kg/m^2^) or obese (≥24 kg/m^2^) (27). Participants were further divided into four groups based on the combination of handgrip strength (two groups: low and normal) and BMI (two groups: normal weight and obese). Those with both obesity and low handgrip strength were defined as having dynapenic obesity. A continuous joint-severity score was calculated as max(Tgrip − observed handgrip strength, 0) + max(BMI − 24, 0), where Tgrip was 26 kg for men and 18 kg for women. The score was treated as exploratory to find the potential dose–response association. Blood pressure was measured by trained healthcare professionals using an electronic sphygmomanometer. Three consecutive measurements were taken at 30-s intervals, and the mean value was used for analysis.

### Statistical analysis

Categorical variables were expressed as frequencies (%), and continuous variables were summarized as medians with interquartile ranges (IQR). Group comparisons were performed using chi-square tests for categorical variables and Mann–Whitney U tests for non-normally distributed continuous variables. Potential multicollinearity was assessed using variance inflation factors (VIFs) after categorical covariates had been expanded into indicator variables. The maximum VIF was 1.98, and all values were below 4.0 (Supplementary Table 1). Variable-level missingness was summarized, and multiple imputation by chained equations with 20 imputed datasets was performed. Logistic regression models were used to estimate crude odds ratios (ORs) and adjusted odds ratios (aORs) with 95% confidence intervals (CIs): Model 1 was unadjusted; Model 2 adjusted for age, sex, education level, married status, smoking status, sleep duration, anxiety disorder; Model 3 additionally included PA and depressive disorders. To assess the robustness of the associations between dynapenic obesity and cognitive disorder, subgroup analyses were conducted by age, sex, married status, smoking status, PA, sleep duration, and social activity. Sensitivity analyses were performed by excluding patients with severe hypertension [systolic blood pressure (SBP) ≥ 200 mmHg and/or diastolic blood pressure (DBP) ≥ 150 mmHg], using an alternative MMSE cutoff (<24 points) for cognitive disorder, excluding severe anxiety or depressive disorders and recorded cerebral infarction or cerebral hemorrhage, using a four-category body-composition phenotype, and modeling PA continuously. For the path analysis, binary endogenous variables were modeled using generalized structural equation modeling with Bernoulli distributions and logit links. As a sensitivity analysis, an observed-variable path model was fitted with physical activity, depressive-symptom score, and MMSE score specified as continuous variables. All equations were adjusted for age, sex, education level, married status, smoking status, sleep duration, anxiety disorder. Log likelihood, AIC, and BIC were reported for model documentation.

All analyses were conducted using Stata/MP 18.0 and Python 3.12.13. A two-tailed *p* < 0.05 was considered statistically significant.

## Results

### The characteristics of participants

A total of 9,568 participants were included in this study, with 42.79% being men and a median age of 72 years. The prevalence of dynapenic obesity and cognitive disorder among the participants was 34.41% (95%CI: 33.45–35.36%) and 15.67% (95%CI: 14.94–16.40%), respectively. Compared to participants with dynapenic obesity, those without dynapenic obesity shown significant differences in age, sex, education level, married status, smoking status, PA, sleep duration, anxiety disorders, depressive disorders, cognitive disorder (all *p* < 0.05). Detailed characteristics stratified by dynapenic obesity are presented in Table 1. The missing-data profile is provided in Supplementary Table 2. The distribution of the main and associated variables are shown in Supplementary Figure 1.

**Table 1:** The characteristic of all participants.

Variables	Total (*n* = 9,568)	Dynapenic obesity		*p*
		Yes (*n* = 3,292)	No (*n* = 6,276)	
Age, years*	72 (69–76)	72 (69–76)	72 (69–76)	<0.001
Sex^				0.018
Men	4,094 (42.79)	1,354 (41.13)	2,740 (43.66)	
Women	5,474 (57.21)	1938 (58.87)	3,536 (56.34)	
Education level^				<0.001
Below Primary school	3,279 (34.27)	1,136 (34.51)	2,143 (34.15)	
Primary school	3,884 (40.59)	1,420 (43.13)	2,464 (39.26)	
Middle school and above	2,405 (25.14)	736 (22.36)	1,669 (26.59)	
Married status^				0.008
Married	7,235 (75.73)	2,542 (77.33)	4,693 (74.88)	
Not married	2,319 (24.27)	745 (22.67)	1,574 (25.12)	
Smoking status^				0.011
Smoking	1,495 (15.64)	464 (14.11)	1,031 (16.45)	
Ex-smoking	1,006 (10.53)	351 (10.67)	655 (10.45)	
Never smoking	7,057 (73.83)	2,474 (75.22)	4,583 (73.11)	
Drinking status^				0.252
No	8,704 (91.25)	3,007 (91.70)	5,697 (91.01)	
Yes	835 (8.75)	272 (8.30)	563 (8.99)	
Physical activity^				<0.001
Lower level	2,458 (25.69)	1,000 (30.38)	1,458 (23.23)	
Appropriate level	5,963 (62.32)	1933 (58.72)	4,030 (64.21)	
Missing	1,147 (11.99)	359 (10.91)	788 (12.56)	
Sleep duration*	7 (6–8)	7 (6-8)	7 (6–8)	<0.001
Social activity^				0.513
No	6,585 (68.87)	2,280 (69.30)	4,305 (68.65)	
Yes	2,976 (31.13)	1,010 (30.70)	1966 (31.35)	
Anxiety disorders^				<0.001
No	7,850 (82.04)	2,637 (80.10)	5,213 (83.06)	
Yes	1718 (17.96)	655 (19.90)	1,063 (16.94)	
Depressive disorders^				<0.001
No	8,694 (90.87)	2,939 (89.28)	5,755 (91.70)	
Yes	874 (9.13)	353 (10.72)	521 (8.30)	
Cognitive disorder^				<0.001
No	8,069 (84.33)	2,670 (81.11)	5,399 (86.03)	
Yes	1,499 (15.67)	622 (18.89)	877 (13.97)	

^*^Data are median (lower quartiles-upper quartiles); ^Data are number (percentage); *p*-value is from Mann–Whitney *U* test or chi-square test for continuous variables and categorical variables.

### The association between dynapenic obesity and cognitive disorder

Among the study participants, 877 participants without dynapenic obesity and 622 participants with dynapenic obesity were diagnosed with cognitive disorder. The prevalence of cognitive disorder was 13.97% (95% CI: 13.12–14.83%) in patients without dynapenic obesity and 18.89% (95% CI: 17.56–20.23%) in those with dynapenic obesity. After adjustment for age, sex, education level, marital status, smoking status, sleep duration, and anxiety disorder, patients with dynapenic obesity had a higher odds ratio of cognitive disorder compared to those without dynapenic obesity. Detailed results are provided in Table 2. After multiple imputation, the association was robust (Supplementary Table 3).

**Table 2:** Subgroup associations between dynapenic obesity and cognitive disorder before multiple imputation.

Variables	Model 1		Model 2		Model 3	
	OR (95%CI)	*p*	aOR (95%CI)	*p*	aOR (95%CI)	*p*
Dynapenic obesity						
No	1.000 (ref.)		1.000 (ref.)		1.000 (ref.)	
Yes	1.434 (1.281–1.605)	<0.001	1.418 (1.263–1.591)	<0.001	0.999 (0.867–1.152)	0.991
Age, years						
<75^#^	1.511 (1.307–1.746)	<0.001	1.519 (1.308–1.763)	<0.001	1.033 (0.855–1.248)	0.734
≥75^#^	1.305 (1.091–1.562)	0.004	1.335 (1.110–1.605)	0.002	0.989 (0.796–1.230)	0.923
Sex						
Men^#^	1.285 (1.081–1.529)	0.005	1.281 (1.073–1.529)	0.006	0.935 (0.754–1.161)	0.543
Women^#^	1.560 (1.344–1.810)	<0.001	1.529 (1.312–1.781)	<0.001	1.054 (0.872–1.274)	0.588
Education level						
Below primary school^#^	1.078 (0.862–1.349)	0.509	1.076 (0.856–1.353)	0.528	0.981 (0.767–1.255)	0.879
Primary school^#^	1.931 (1.626–2.293)	<0.001	1.779 (1.491–2.123)	<0.001	1.106 (0.858–1.427)	0.437
Middle school or above^#^	1.276 (1.034–1.574)	0.023	1.250 (1.009–1.549)	0.041	0.988 (0.774–1.260)	0.920
Married status						
Married^#^	1.605 (1.412–1.825)	<0.001	1.551 (1.359–1.770)	<0.001	1.017 (0.858–1.205)	0.848
Not married^#^	0.985 (0.773–1.254)	0.901	1.021 (0.797–1.308)	0.870	0.968 (0.747–1.256)	0.809
Smoking status						
Smoking^#^	1.898 (1.445–2.494)	<0.001	1.638 (1.228–2.185)	<0.001	1.239 (0.804–1.910)	0.331
Ex-smoking^#^	0.962 (0.649–1.426)	0.848	0.922 (0.615–1.383)	0.696	0.855 (0.564–1.294)	0.458
Never smoking^#^	1.418 (1.244–1.617)	<0.001	1.407 (1.231–1.609)	<0.001	0.991 (0.842–1.165)	0.909
Drinking status						
No^#^	1.426 (1.268–1.604)	<0.001	1.417 (1.257–1.598)	<0.001	1.010 (0.874–1.168)	0.894
Yes^#^	1.507 (1.003–2.264)	0.048	1.468 (0.943–2.286)	0.089	0.673 (0.290–1.562)	0.357
Physical activity						
Inadequate^#^	0.957 (0.778–1.178)	0.679	1.001 (0.806–1.245)	0.989	0.996 (0.801–1.238)	0.970
Adequate^#^	0.995 (0.826–1.199)	0.960	1.041 (0.862–1.259)	0.674	1.041 (0.861–1.258)	0.678
Sleep duration, hours						
<7^#^	1.788 (1.512–2.114)	<0.001	1.699 (1.428–2.021)	<0.001	1.183 (0.921–1.520)	0.187
≥7^#^	1.094 (0.933–1.282)	0.268	1.129 (0.960–1.327)	0.141	0.923 (0.775–1.098)	0.364
Social activity						
No^#^	1.711 (1.491–1.964)	<0.001	1.673 (1.451–1.929)	<0.001	1.067 (0.878–1.297)	0.514
Yes^#^	1.009 (0.826–1.231)	0.932	1.013 (0.824–1.244)	0.903	0.976 (0.790–1.206)	0.824
Anxiety disorders						
No^#^	1.461 (1.284–1.663)	<0.001	1.462 (1.282–1.668)	<0.001	0.963 (0.815–1.137)	0.653
Yes^#^	1.276 (1.010–1.612)	0.041	1.284 (1.010–1.632)	0.041	1.112 (0.844–1.466)	0.450
Depressive disorders						
No^#^	1.466 (1.301–1.653)	<0.001	1.461 (1.292–1.652)	<0.001	0.984 (0.843–1.149)	0.841
Yes^#^	1.127 (0.807–1.573)	0.482	1.096 (0.771–1.557)	0.610	1.075 (0.748–1.546)	0.695

Model 1 was unadjusted. Model 2 was adjusted for age, sex, education level, marital status, smoking status, sleep duration, and anxiety disorders. Model 3 additionally included physical activity and depressive disorders. OR, odds ratio; aOR, adjusted odds ratio; CI, confidence interval. ^#^The reference group was no dynapenic obesity group.

The continuous joint-severity score was also positively and linear associated with cognitive disorder (*P*_non-linear_ = 0.181). The aOR = 1.085 (95% CI: 1.075–1.096) per one-unit increase, 1.505 (95% CI: 1.435–1.579) per five-unit increase, and 1.626 (95% CI: 1.536–1.720) per one-SD increase.

### Subgroup analysis results of the association between dynapenic obesity and cognitive disorder

In model 2, subgroup analyses by age and sex groups confirmed the robustness of the association of dynapenic obesity and cognitive disorder (*p* < 0.05). Additionally, significant associations of dynapenic obesity and cognitive disorder were also observed among participants with primary-school or higher education, married participants, current or never smokers, non-drinkers, those sleeping <7 h, those who did not participate in social activities, participants with or without anxiety disorder, and those without depressive disorders (all *p* < 0.05). Further details are available in Table 2.

After multiple imputation, the associations of dynapenic obesity and cognitive disorder remained statistically significant in both age and sex groups, and among participants with primary-school education, married participants, current or never smokers, non-drinkers, those with an appropriate level of physical activity, those sleeping <7 h, those who did not participate in social activities, participants with or without anxiety disorder, and those without depressive disorders (all *p* < 0.05). Further details are available in Supplementary Table 3.

### Sensitivity analysis results of this association

The association between dynapenic obesity and cognitive disorder remained robust after excluding patients with severe hypertension. Compared to patients without dynapenic obesity, those with dynapenic obesity had a higher risk of cognitive disorder (aOR = 1.424, 95% CI: 1.268–1.598). Furthermore, when the cut-off point for identifying cognitive disorder changed to 24 points, the association persisted (aOR = 1.325, 95% CI: 1.205–1.456). After participants with recorded cerebral infarction or cerebral hemorrhage were excluded, the confounder-adjusted association remained positive (aOR = 1.451, 95% CI: 1.273–1.654). Excluding participants with severe anxiety or severe depressive disorders an aOR of 1.423 (95% CI: 1.269–1.597), and applying both exclusion criteria an aOR of 1.439 (95% CI: 1.262–1.641). Using low handgrip strength together with waist-defined abdominal obesity produced an aOR of 1.748 (95% CI: 1.557–1.963). After multiple imputation, the results were robust. Details are shown in Supplementary Table 4.

### Path analysis results of this association

Dynapenic obesity was associated with lower odds of adequate physical activity (OR = 0.708, 95% CI: 0.640–0.783), whereas its conditional association with cognitive disorder was close to null (OR = 0.999, 95% CI: 0.867–1.152). Adequate physical activity was associated with lower odds of depressive disorders (OR = 0.441, 95% CI: 0.367–0.529) and cognitive disorder (OR = 0.521, 95% CI: 0.452–0.601). The path from dynapenic obesity to depressive disorders was not statistically significant (OR = 1.133, 95% CI: 0.944–1.360), whereas depressive disorders were associated with higher odds of cognitive disorder (OR = 1.312, 95% CI: 1.031–1.669). The conditional direct coefficient was −0.001 (95% CI: −0.143 to 0.141). On the coefficient scale, the product-of-coefficients estimate through physical activity was 0.225 (95% simulation interval: 0.148–0.313), the estimate through depressive disorders was 0.034 (95% simulation interval: −0.015 to 0.109), and the physical activity–depressive disorder pathway was 0.077 (95% simulation interval: 0.008–0.159). The coefficient-scale total was 0.335 (95% simulation interval: 0.145–0.543). Details are shown in Figure 2 and Supplementary Tables 5A,B. After multiple imputation, path estimates and uncertainty intervals are reported in Supplementary Figure 2 and Supplementary Tables 6A,B. Supplementary Table 7 are comparison of the complete-case and multiply imputed path models. The results of sensitivity analysis fitted with physical activity, depressive-symptom score, and MMSE score specified as continuous variables were robust (Supplementary Figure 3 and Supplementary Tables 8A,B).

**Figure 2:**
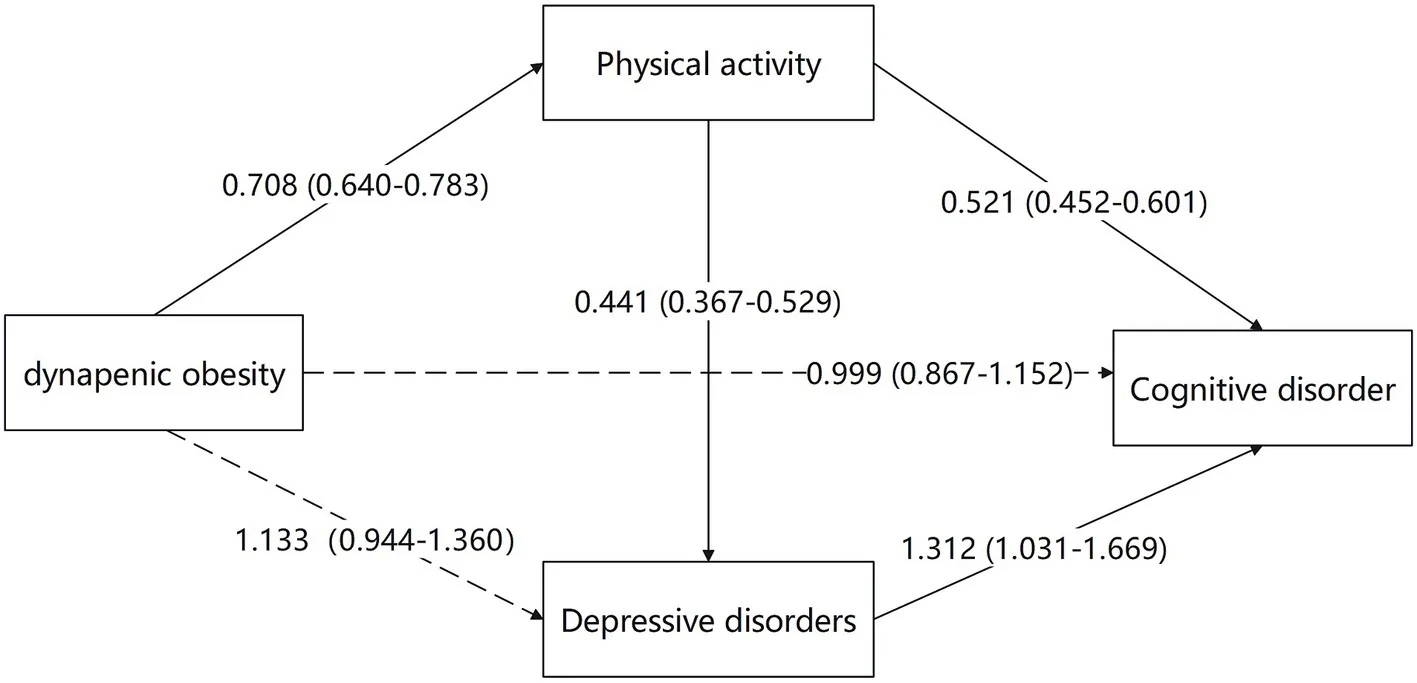
The results of path model before multiple imputation. Adjusted for age, sex, education level, marital status, smoking status, sleep duration, and anxiety disorders. Solid arrows indicate statistically significant paths, whereas dashed arrows indicate non-significant paths.

## Discussion

This multicenter cross-sectional study yielded three principal findings regarding cognitive disorder among the older HTN patients. First, we identified a substantial prevalence rate of 15.67% for cognitive disorder in this population. Second, we demonstrated a significant positive association between dynapenic obesity and cognitive disorder, with this association maintaining robust consistency across all age-stratified subgroups. Third, our path analysis revealed that PA, depressive disorders, dynapenic obesity, and cognitive disorder were related within a hypothesized generalized path model.

The prevalence of cognitive disorder was 15.67% among the older HTN individuals, indicating a substantial burden. Because no normotensive comparison group was included, this estimate should not be interpreted as significantly higher than that of the general population (28). Evidence suggests three pathways appear particularly consequential: (1) chronic cerebral hypoperfusion that induces white matter lesions and microstructural damage (29), (2) blood–brain barrier dysfunction that facilitates neuroinflammatory processes (30), and (3) impaired vascular clearance mechanisms that promote amyloid-*β* deposition (31). These neuropathological insights underscore the critical need for a paradigm shift in HTN management. Specifically, we recommend implementing routine cognitive screening within standard HTN care protocols, developing integrated care models that simultaneously address cardiovascular and neurocognitive health, and designing targeted interventions focused on modifiable risk factors.

Our study identified a positive association between dynapenic obesity and cognitive disorder among older patients with hypertension, consistent with findings in general aging populations (9, 10, 32, 33). Oba et al. (12) used waist circumference among patients with cardiometabolic disease, Smith et al. (11) studied community-dwelling adults across multiple countries, and Zhu et al. (34) provided longitudinal evidence on cognitive trajectories. These studies supported evaluation of combined muscle weakness and adiposity but did not make their effect estimates directly interchangeable with the present BMI-based definition.

In the present hypertensive population, dynapenic obesity was associated with higher odds of cognitive disorder in the complete-case, confounder-adjusted analysis (aOR = 1.418, 95% CI: 1.263–1.591). In comparison, a community-based study of 32,715 adults aged ≥50 years from six low- and middle-income countries reported an adjusted OR of 1.45 (95% CI: 1.11–1.88) for mild cognitive disorder among participants with dynapenic abdominal obesity; in the subgroup aged ≥65 years, the association was smaller and not statistically significant (OR = 1.29, 95% CI: 0.92–1.80) (11). These between-study differences should not be interpreted as evidence that hypertension amplified the association because phenotype definitions, cognitive outcomes, covariate sets, and source populations differed, and the present study included no normotensive comparison group. Other studies in general aging and cardiometabolic populations likewise supported evaluation of combined muscle weakness and adiposity, but their estimates were not directly interchangeable with the present BMI-based definition (9, 10, 12, 32–34). The positive association in both age subgroups suggested that the finding was not restricted to one prespecified age stratum. However, subgroup significance did not establish an independent or causal effect. The sensitivity analyses supported consistency under the evaluated assumptions, but residual confounding and reverse causation could not be excluded. Longitudinal studies are needed to assess temporality and whether changes in dynapenic obesity are followed by changes in cognition.

Dynapenic obesity was associated with lower odds of adequate physical activity, whereas adequate physical activity was associated with lower odds of both depressive disorders and cognitive disorder. The conditional association between dynapenic obesity and cognitive disorder was close to null after physical activity and depressive disorders had been incorporated as endogenous pathway variables. This pattern was compatible with the possibility that physical-activity-related processes contributed to the observed association, although statistical mediation and temporal ordering could not be established. Several biologically plausible mechanisms may explain the associations involving physical activity, including reduced cerebral perfusion and chronic hippocampal hypoperfusion (35), diminished production of brain-derived neurotrophic factor and other neurotrophic factors required for synaptic plasticity (36), and increased systemic inflammation characterized by elevated pro-inflammatory cytokines, such as soluble tumor necrosis factor-*α* receptor 1 (37).

Previous cross-sectional and prospective studies showed that dynapenic obesity was associated with impaired mobility, deterioration in physical functioning, and the subsequent onset of disability in activities of daily living (38–40). Conversely, randomized studies indicated that physical activity and structured exercise could preserve muscle strength, limit muscle fat infiltration, and improve physical functioning in older adults, including those with obesity (41, 42). These findings supported the plausibility of reciprocal associations between dynapenic obesity and PA but did not establish the temporal direction of the association examined in this cross-sectional study.

Depressive disorders were positively associated with cognitive disorder; however, the path from dynapenic obesity to depressive disorders and the separate coefficient-scale pathway through depressive disorders were not statistically significant. Depressive disorders should therefore be interpreted as a related neuropsychiatric correlate rather than an established mediator of the association. Potential mechanisms linking depressive disorders with cognitive disorder may include dysregulation of the hypothalamic–pituitary–adrenal axis and prolonged cortisol exposure (43), disturbances in sleep architecture involving reduced slow-wave sleep (44), and social withdrawal accompanied by reduced cognitive stimulation (45). These findings provided hypothesis-generating support for an integrated biopsychosocial framework. Future longitudinal and intervention studies should determine whether strategies addressing physical inactivity, depressive disorders, sleep disturbances, and limited social engagement can reduce subsequent cognitive disorder among individuals with dynapenic obesity.

Our study possesses several noteworthy strengths. Firstly, it included a large multicenter sample in China to systematically evaluate the prevalence of cognitive disorder among the older HTN patients, while simultaneously elucidating the path of PA and depressive disorders in the association between dynapenic obesity and cognitive disorder within this specific population. Secondly, our findings highlight the potential value of routine cognitive screening among patients with hypertension and dynapenic obesity, together with integrated care approaches addressing metabolic and mental health. Thirdly, we used well-established and psychometrically validated instruments for screening depressive disorders and cognitive disorder, which have demonstrated robust reliability and validity across diverse Chinese populations.

However, several limitations warrant careful consideration when interpreting our findings. The cross-sectional design fundamentally precludes causal inference regarding the observed associations between dynapenic obesity and cognitive disorder. PA had 11.99% missingness, although multiple imputation was performed under MAR. Despite comprehensive adjustment for known covariates, potential confounding from unmeasured variables persists. For example, we lacked data on genetic risk factors, which could influence cognitive condition. Our definition of dynapenic obesity, while standardized, may not fully capture the biological complexity of this condition. The reliance on handgrip strength and BMI, overlooks regional body composition differences (e.g., visceral vs. subcutaneous fat). measurement via self-report could introduce recall bias. Dichotomizing dynapenic obesity, PA, depressive disorders, and cognitive disorder may have reduced statistical information and obscured dose–response patterns; the continuous-score analyses were exploratory and did not eliminate this limitation. Because dynapenic obesity, PA, depressive disorders, and cognitive status were assessed concurrently, temporal precedence could not be determined. Dynapenic obesity may have restricted PA through impaired strength and mobility; conversely, insufficient PA may have contributed to muscle weakness and adiposity, and cognitive disorder may itself have reduced activity participation. Reverse causation and reciprocal associations therefore could not be excluded, and the indirect pathways should not be interpreted as causal mediation effects. Finally, restricting the sample to older adults with hypertension improved clinical specificity but limited generalizability and did not permit hypertension to be evaluated as an effect modifier.

## Conclusion

The study found that the prevalence of cognitive disorder among older patients with hypertension was 15.67%. Dynapenic obesity was positively associated with cognitive disorder. PA and depressive disorders were related within a hypothesized cross-sectional path structure; however, causal mediation could not be established. Longitudinal studies are required before intervention effects can be inferred.

## Data Availability

The data analyzed in this study is subject to the following licenses/restrictions: Data will be made available on request. Requests to access these datasets should be directed to liqm@zzu.edu.cn.

## Author’s note

All authors confirm that the following manuscript is a transparent and honest account of the reported research. This research is related to a previous study by the same authors titled “Association between healthy lifestyle and the risk of ischemic stroke among elderly adults with hypertension: a cross-sectional study in China”. The same study population was used in both articles. The previous study was performed on exploring the association between lifestyle behaviors and ischemic stroke among older adults with hypertension, and the current submission is focusing on examining the prevalence of cognitive disorder in this population, assessing the association between dynapenic obesity and cognitive disorder, and further examining a cross-sectional path structure involving physical activity and depressive disorders. The study is following the methodology explained in Methods section.
