# Supplementary material for: The associations between dynapenic obesity and cognitive disorder in older hypertension patients: a multicenter cross-sectional study
Source: Front Public Health. 2026 Sep 29;14:1893791. doi: 10.3389/fpubh.2026.1893791 (PMC13657995; doi:10.3389/fpubh.2026.1893791)
Supplement: Supplementary file 1 [file Data_Sheet_1.docx]

STROBE Statement—Checklist of items that should be included in reports of ***cross-sectional studies***

|  | Item No | Recommendation | Page No |
| --- | --- | --- | --- |
| **Title and abstract** | 1 | (*a*) Indicate the study’s design with a commonly used term in the title or the abstract | 1 |
|  |  | (*b*) Provide in the abstract an informative and balanced summary of what was done and what was found | 1 |
| Introduction | | | |
| Background/rationale | 2 | Explain the scientific background and rationale for the investigation being reported | 2 |
| Objectives | 3 | State specific objectives, including any prespecified hypotheses | 2 |
| Methods | | | |
| Study design | 4 | Present key elements of study design early in the paper | 2 |
| Setting | 5 | Describe the setting, locations, and relevant dates, including periods of recruitment, exposure, follow-up, and data collection | 2,3 |
| Participants | 6 | (*a*) Give the eligibility criteria, and the sources and methods of selection of participants | 2,3 |
| Variables | 7 | Clearly define all outcomes, exposures, predictors, potential confounders, and effect modifiers. Give diagnostic criteria, if applicable | 2,3 |
| Data sources/ measurement | 8* | For each variable of interest, give sources of data and details of methods of assessment (measurement). Describe comparability of assessment methods if there is more than one group | 2,3 |
| Bias | 9 | Describe any efforts to address potential sources of bias | 3 |
| Study size | 10 | Explain how the study size was arrived at | 2 |
| Quantitative variables | 11 | Explain how quantitative variables were handled in the analyses. If applicable, describe which groupings were chosen and why | 2,3 |
| Statistical methods | 12 | (*a*) Describe all statistical methods, including those used to control for confounding | 3,4 |
|  |  | (*b*) Describe any methods used to examine subgroups and interactions | 3 |
|  |  | (*c*) Explain how missing data were addressed | 3 |
|  |  | (*d*) If applicable, describe analytical methods taking account of sampling strategy | N/A |
|  |  | (*e*) Describe any sensitivity analyses | 3 |
| Results | | | |
| Participants | 13* | (a) Report numbers of individuals at each stage of study—eg numbers potentially eligible, examined for eligibility, confirmed eligible, included in the study, completing follow-up, and analysed | 2, Figure 1 |
|  |  | (b) Give reasons for non-participation at each stage | Figure 1 |
|  |  | (c) Consider use of a flow diagram | Figure 1 |
| Descriptive data | 14* | (a) Give characteristics of study participants (eg demographic, clinical, social) and information on exposures and potential confounders | 2,3, Table 1 |
|  |  | (b) Indicate number of participants with missing data for each variable of interest | Supplementary Table 2 |
| Outcome data | 15* | Report numbers of outcome events or summary measures | 4 |
| Main results | 16 | (*a*) Give unadjusted estimates and, if applicable, confounder-adjusted estimates and their precision (eg, 95% confidence interval). Make clear which confounders were adjusted for and why they were included | Table 2 Supplementary Table 3 |
|  |  | (*b*) Report category boundaries when continuous variables were categorized | 2,3 |
|  |  | (*c*) If relevant, consider translating estimates of relative risk into absolute risk for a meaningful time period | N/A |
| Other analyses | 17 | Report other analyses done—eg analyses of subgroups and interactions, and sensitivity analyses | 4 |
| Discussion | | | |
| Key results | 18 | Summarise key results with reference to study objectives | 5 |
| Limitations | 19 | Discuss limitations of the study, taking into account sources of potential bias or imprecision. Discuss both direction and magnitude of any potential bias | 8 |
| Interpretation | 20 | Give a cautious overall interpretation of results considering objectives, limitations, multiplicity of analyses, results from similar studies, and other relevant evidence | 5-8 |
| Generalisability | 21 | Discuss the generalisability (external validity) of the study results | 8 |
| Other information | | | |
| Funding | 22 | Give the source of funding and the role of the funders for the present study and, if applicable, for the original study on which the present article is based | 8 |

*Give information separately for exposed and unexposed groups.

**Note:** An Explanation and Elaboration article discusses each checklist item and gives methodological background and published examples of transparent reporting. The STROBE checklist is best used in conjunction with this article (freely available on the Web sites of PLoS Medicine at http://www.plosmedicine.org/, Annals of Internal Medicine at http://www.annals.org/, and Epidemiology at http://www.epidem.com/). Information on the STROBE Initiative is available at www.strobe-statement.org.

**Supplementary Table 1**. Variance inflation factors for covariates included in the adjusted model.

| **Variables** | **VIF** |
| --- | --- |
| Age, years* | 1.12 |
| Sex^ |  |
| Men | Ref. |
| Women | 1.58 |
| Education level^ |  |
| Below primary school | Ref. |
| Primary school | 1.32 |
| Middle school and above | 1.46 |
| Married status^ |  |
| Married | Ref. |
| Not married | 1.09 |
| Smoking status^ |  |
| Smoking | Ref. |
| Ex-smoking | 1.61 |
| Never smoking | 1.98 |
| Physical activity^ |  |
| Lower level | Ref. |
| Appropriate level | 1.06 |
| Sleep duration* | 1.06 |
| Anxiety disorders^ |  |
| No | Ref. |
| Yes | 1.48 |
| Depressive disorders^ |  |
| No | Ref. |
| Yes | 1.48 |

**Supplementary Table 2.** Distribution of missing data and comparison of participant characteristics between complete and incomplete cases.

| **Variables** | **Missing, n** | **Missing, %** | ***P*** |
| --- | --- | --- | --- |
| Age | 32 | 0.33% | 0.394 |
| Sex | 0 | 0.00% | —— |
| Education level | 0 | 0.00% | —— |
| Married status | 14 | 0.15% | <0.001 |
| Smoking status | 10 | 0.10% | <0.001 |
| Drinking status | 29 | 0.30% | <0.001 |
| **Physical activity** | **1,147** | **11.99%** | 0.051 |
| Sleep duration | 29 | 0.30% | <0.001 |
| Social activity | 7 | 0.07% | <0.001 |
| Anxiety disorders | 5 | 0.05% | 0.060 |
| Depressive disorders | 0 | 0.00% | —— |
| Dynapenic Obesity | 0 | 0.00% | —— |
| Cognitive disorder | 0 | 0.00% | —— |

**Supplementary Table 3.** Subgroup associations between dynapenic obesity and cognitive disorder after multiple imputation.

| **Variables** | **Model 1** | | **Model 2** | | **Model 3** | |
| --- | --- | --- | --- | --- | --- | --- |
|  | **OR(95%CI)** | ***P*** | **aOR(95%CI)^*^** | ***P*** | **aOR(95%CI)^*^** | ***P*** |
| Dynapenic Obesity |  |  |  |  |  |  |
| No | 1.000(ref.) |  | 1.000(ref.) |  | 1.000(ref.) |  |
| Yes | 1.434 (1.281-1.605) | <0.001 | 1.411 (1.258-1.583) | <0.001 | 1.352 (1.203-1.519) | <0.001 |
| Age, years |  |  |  |  |  |  |
| <75^#^ | 1.509 (1.305-1.744) | <0.001 | 1.509 (1.301-1.751) | <0.001 | 1.442 (1.240-1.676) | <0.001 |
| ≥75^#^ | 1.306 (1.091-1.563) | 0.004 | 1.329 (1.106-1.597) | 0.002 | 1.282 (1.064-1.545) | 0.009 |
| Sex |  |  |  |  |  |  |
| Men^#^ | 1.285 (1.081-1.529) | 0.005 | 1.270 (1.065-1.516) | 0.008 | 1.214 (1.015-1.452) | 0.034 |
| Women^#^ | 1.560 (1.344-1.810) | <0.001 | 1.526 (1.310-1.778) | <0.001 | 1.464 (1.255-1.708) | <0.001 |
| Education level |  |  |  |  |  |  |
| Below primary school^#^ | 1.078 (0.862-1.349) | 0.509 | 1.070 (0.851-1.344) | 0.563 | 1.051 (0.833-1.326) | 0.675 |
| Primary school^#^ | 1.931 (1.626-2.293) | <0.001 | 1.782 (1.493-2.126) | <0.001 | 1.780 (1.488-2.130) | <0.001 |
| Middle school or above^#^ | 1.276 (1.034-1.574) | 0.023 | 1.234 (0.996-1.527) | 0.054 | 1.135 (0.912-1.411) | 0.256 |
| Married status |  |  |  |  |  |  |
| Married^#^ | 1.602 (1.410-1.822) | <0.001 | 1.540 (1.350-1.756) | <0.001 | 1.469 (1.286-1.679) | <0.001 |
| Not married^#^ | 0.983 (0.772-1.252) | 0.889 | 1.021 (0.797-1.309) | 0.866 | 0.993 (0.774-1.275) | 0.958 |
| Smoking status |  |  |  |  |  |  |
| Smoking^#^ | 1.897 (1.444-2.492) | <0.001 | 1.636 (1.227-2.182) | <0.001 | 1.623 (1.212-2.172) | 0.001 |
| Ex-smoking^#^ | 0.960 (0.648-1.423) | 0.841 | 0.910 (0.607-1.364) | 0.649 | 0.864 (0.574-1.301) | 0.484 |
| Never smoking^#^ | 1.419 (1.245-1.618) | <0.001 | 1.402 (1.226-1.602) | <0.001 | 1.330 (1.162-1.523) | <0.001 |
| Drinking status |  |  |  |  |  |  |
| No^#^ | 1.426 (1.268-1.604) | <0.001 | 1.408 (1.249-1.587) | <0.001 | 1.343 (1.190-1.517) | <0.001 |
| Yes^#^ | 1.513 (1.008-2.273) | 0.046 | 1.464 (0.942-2.277) | 0.090 | 1.444 (0.927-2.250) | 0.104 |
| Physical activity |  |  |  |  |  |  |
| Inadequate^#^ | 1.220 (1.001-1.487) | 0.048 | 1.204 (0.984-1.472) | 0.071 | 1.210 (0.989-1.480) | 0.064 |
| Adequate^#^ | 1.478 (1.264-1.728) | <0.001 | 1.503 (1.282-1.762) | <0.001 | 1.502 (1.282-1.761) | <0.001 |
| Sleep duration, hours |  |  |  |  |  |  |
| <7^#^ | 1.784 (1.509-2.109) | <0.001 | 1.688 (1.419-2.008) | <0.001 | 1.641 (1.375-1.959) | <0.001 |
| ≥7^#^ | 1.096 (0.935-1.284) | 0.257 | 1.124 (0.956-1.321) | 0.157 | 1.076 (0.915-1.267) | 0.375 |
| Social activity |  |  |  |  |  |  |
| No^#^ | 1.712 (1.491-1.964) | <0.001 | 1.672 (1.450-1.927) | <0.001 | 1.618 (1.400-1.870) | <0.001 |
| Yes^#^ | 1.009 (0.827-1.232) | 0.929 | 1.000 (0.815-1.228) | 0.998 | 0.950 (0.772-1.171) | 0.633 |
| Anxiety disorder |  |  |  |  |  |  |
| No^#^ | 1.460 (1.283-1.662) | <0.001 | 1.454 (1.275-1.658) | <0.001 | 1.399 (1.225-1.598) | <0.001 |
| Yes^#^ | 1.277 (1.011-1.614) | 0.040 | 1.279 (1.007-1.624) | 0.044 | 1.214 (0.952-1.549) | 0.118 |
| Depressive disorders |  |  |  |  |  |  |
| No^#^ | 1.466 (1.301-1.653) | <0.001 | 1.454 (1.286-1.644) | <0.001 | 1.398 (1.235-1.583) | <0.001 |
| Yes^#^ | 1.127 (0.807-1.573) | 0.482 | 1.099 (0.775-1.559) | 0.597 | 1.027 (0.720-1.464) | 0.883 |

*Notes.* Model 1 was unadjusted. Model 2 was adjusted for age, sex, education level, marital status, smoking status, sleep duration, and anxiety disorder. Model 3 additionally included physical activity and depressive disorders.

OR, odds ratio; aOR, adjusted odds ratio; CI, confidence interval

^#^The reference group was no dynapenic obesity group.

**Supplementary Table 4.** Sensitivity analyses of dynapenic obesity and cognitive disorder.

|  | Before  multiple imputation | After  multiple imputation |
| --- | --- | --- |
|  | aOR(95% CI) | aOR(95% CI) |
| Excluding patients with severe hypertension | 1.424(1.268-1.598) | 1.416 (1.262-1.590) |
| Using the cut-off point for identifying cognitive disorder changed to 24 points | 1.325(1.205-1.456) | 1.318 (1.200-1.448) |
| Excluding participants with stroke or other major neurological diseases | 1.451(1.273-1.654) | 1.443 (1.264-1.647) |
| Excluding participants with severe depression or anxiety | 1.423(1.269-1.597) | 1.416 (1.261-1.590) |
| Evaluating alternative definitions of dynapenic obesity | 1.748(1.557-1.963) | 1.752 (1.558-1.971) |

Adjusted age, sex, education, marital status, smoking, sleep duration, and anxiety symptoms.

Missing covariates were imputed using multiple imputation by chained equations with 20 imputed datasets.

**Supplementary Table 5A.** Complete-case path estimates

| **Modeled path** | **b** | **OR (95% CI)** | **P** |
| --- | --- | --- | --- |
| Dynapenic obesity — adequate PA | -0.345 | 0.708(0.640-0.783) | <0.001 |
| Dynapenic obesity — depressive disorders | 0.125 | 1.133 (0.944-1.360) | 0.181 |
| Adequate PA — depressive disorders | -0.820 | 0.441 (0.367-0.529) | <0.001 |
| Dynapenic obesity — cognitive disorder | -0.001 | 0.999 (0.867-1.152) | 0.991 |
| Adequate PA — cognitive disorder | -0.652 | 0.521 (0.452-0.601) | <0.001 |
| Depressive disorders — cognitive disorder | 0.272 | 1.312 (1.031-1.669) | 0.027 |

b, the unstandardized logit coefficient.

ORs, exponentiated coefficients.

Robust standard errors and Wald 95% confidence intervals were obtained from the Bernoulli-logit generalized path model.

**Supplementary Table 5B.** Complete-case coefficient-scale direct, indirect, serial, and total quantities

| **Coefficient-scale quantity** | **Estimate** | **95% interval** |
| --- | --- | --- |
| Conditional direct | -0.001 | -0.143 to 0.141 |
| Through physical activity | 0.225 | 0.148 to 0.313 |
| Through depressive disorders | 0.034 | -0.015 to 0.109 |
| Serial PA-depressive disorders pathway | 0.077 | 0.008 to 0.159 |
| Coefficient-scale total | 0.335 | 0.145 to 0.543 |

The direct-effect interval is the robust Wald interval from StataMP 18.

Product and total intervals are 95% parameter-simulation intervals obtained in Python 3.12.13 from 50,000 multivariate-normal coefficient draws.

They are not BCa bootstrap intervals, odds ratios, proportions mediated, or causal effects.

**Supplementary Table 6A.** Multiply imputed path estimates

| **Modeled path** | **b** | **OR (95% CI)** | **P** |
| --- | --- | --- | --- |
| Dynapenic obesity — adequate PA | -0.387 | 0.679 (0.614-0.751) | <0.001 |
| Dynapenic obesity — depressive disorders | 0.122 | 1.130 (0.949-1.344) | 0.169 |
| Adequate PA — depressive disorders | -0.734 | 0.480 (0.400-0.575) | <0.001 |
| Dynapenic obesity — cognitive disorder | 0.278 | 1.321 (1.175-1.485) | <0.001 |
| Adequate PA — cognitive disorder | -0.803 | 0.448 (0.389-0.517) | <0.001 |
| Depressive disorders — cognitive disorder | -0.221 | 0.802 (0.639-1.006) | 0.056 |

b denotes the unstandardized logit coefficient, calculated as the natural logarithm of the reported OR. ORs and confidence intervals were pooled across 20 imputed datasets.

**Supplementary Table 6B.** Multiply imputed coefficient-scale direct, indirect, serial, and total quantities

| **Coefficient-scale quantity** | **Estimate** | **95% interval** |
| --- | --- | --- |
| Conditional direct | 0.278 | 0.158 to 0.395 |
| Through physical activity | 0.312 | 0.210 to 0.416 |
| Through depressive disorders | -0.027 | -0.089 to 0.013 |
| Serial PA-depressive-symptom pathway | -0.063 | -0.140 to 0.001 |
| Coefficient-scale total | 0.500 | 0.332 to 0.665 |

Coefficient-scale quantities are scale dependent and were not interpreted as conventional odds ratios, proportions mediated, or causal effects.

**Supplementary Table 7.** Comparison of the complete-case and multiply imputed path models

| **Characteristic** | **Before imputation** | **After multiple imputation** |
| --- | --- | --- |
| Analysis sample | 8,334 | 9,568 |
| Conditional DO — cognitive disorder | 0.999 (0.867-1.152) | 1.321 (1.175-1.485) |
| Coefficient-scale direct estimate | -0.001 (-0.143 to 0.141) | 0.278 (0.158-0.395) |
| Coefficient-scale total | 0.335 (0.145-0.543) | 0.500 (0.332-0.665) |
| Log pseudolikelihood | -9,359.872 | — |
| AIC | 18,791.74 | — |
| BIC | 19,044.76 | — |

All model equations were adjusted for age, sex, education level, marital status, smoking status, sleep duration, and anxiety disorder.

PA, physical activity; OR, odds ratio; CI, confidence interval; AIC, Akaike information criterion; BIC, Bayesian information criterion.

**Supplementary Table 8A.** The sensitivity path analysis

| **Modeled path** | **b** | **Standardized β (95% CI)** | **P** |
| --- | --- | --- | --- |
| Dynapenic obesity — PA | -1.873 | -0.085 (-0.104 to -0.066) | <0.001 |
| Dynapenic obesity — depressive disorders | 0.091 | 0.013 (-0.004 to 0.030) | 0.130 |
| PA — depressive disorders | -0.019 | -0.060 (-0.077 to -0.043) | <0.001 |
| Dynapenic obesity — MMSE | -0.358 | -0.033 (-0.052 to -0.013) | 0.001 |
| PA — MMSE | 0.040 | 0.081 (0.064 to 0.098) | <0.001 |
| Depressive disorders — MMSE | -0.212 | -0.134 (-0.163 to -0.104) | <0.001 |

PA, depressive disorders, and MMSE scores were treated as continuous variables in the sensitivity path analysis.

**Supplementary Table 8B.** The coefficient-scale direct, indirect, serial, and total quantities of sensitivity path analysis

| **Coefficient-scale quantity** | **Estimate** | **95% interval** |
| --- | --- | --- |
| Conditional direct | -0.358 | -0.574 to -0.142 |
| Through physical activity | -0.075 | -0.097 to -0.053 |
| Through depressive disorders | -0.019 | -0.044 to 0.006 |
| physical activity-depressive disorders pathway | -0.008 | -0.011 to -0.004 |
| Coefficient-scale total | -0.460 | -0.677 to -0.242 |

**Supplementary Figure 1.** The distribution of dynapenic obesity, cognitive disorder, physical activity and depression disorder.

The data of physical activity were missing for 1,147 participants (11.99%).

**Supplementary Figure 2.** Generalized path model after multiple imputation

Adjusted for age, sex, education level, marital status, smoking status, sleep duration, and anxiety disorders.

Twenty imputations were generated using multiple imputation by chained equations. Estimates were combined using Rubin's rules.

Solid arrows indicate statistically significant paths, whereas dashed arrows indicate non-significant paths.

**Supplementary Figure 3.** The sensitivity path analysis

Adjusted for age, sex, education level, marital status, smoking status, sleep duration, and anxiety disorders.

Solid arrows indicate statistically significant paths, whereas dashed arrows indicate non-significant paths.
